# Comparison of Patient Outcomes following Implantation of Trifocal and Extended Depth of Focus Intraocular Lenses: A Systematic Review and Meta-Analysis

**DOI:** 10.1155/2021/1115076

**Published:** 2021-12-29

**Authors:** Yining Guo, Yinhao Wang, Ran Hao, Xiaodan Jiang, Ziyuan Liu, Xuemin Li

**Affiliations:** ^1^Department of Ophthalmology, Peking University Third Hospital, Beijing 100191, China; ^2^Beijing Key Laboratory of Restoration of Damaged Ocular Nerve, Beijing 100191, China

## Abstract

**Purpose:**

The purpose is to compare the outcomes of implantation of trifocal intraocular lenses (TIOLs) and extended depth of focus (EDOF) intraocular lenses (IOLs).

**Methods:**

A comprehensive search of PubMed, Cochrane Library, EMBASE, and ClinicalTrial.gov was conducted in March 2020 to identify relevant studies. A meta-analysis of the results was performed. Patients implanted with EDOF IOLs or TIOLs in previous studies were included. The primary outcomes of the study were uncorrected distance visual acuity (UDVA), uncorrected intermediate visual acuity (UIVA), uncorrected near visual acuity (UNVA), and defocus curve.

**Results:**

TIOLs and EDOF IOLs provided comparable binocular UDVA (MD = -0.01, 95% CI: -0.04, 0.03, logMAR). However, EDOF IOLs provided better UIVA (MD: -0.08, 95% CI: -0.14, -0.01, logMAR) and worse UNVA (MD: 0.10, 95% CI: 0.06, 0.14, logMAR) than TIOLs. Fewer patients achieved spectacle independence after implantation of EDOF IOLs (RR: 0.74, 95% CI: 0.63, 0.87) than after implantation of TIOLs, especially for near vision (RR = 0.82, 95% CI: 0.68, 0.99). There was no statistically significant difference in contrast sensitivity (CS) under photopic or mesopic conditions with both IOLs. Patient satisfaction after implantation of both IOLs was high.

**Conclusion:**

EDOF IOLs and TIOLs provide comparable distance vision. However, EDOF IOLs provide better intermediate vision and worse near vision than TIOLs. The advantages of EDOF IOLs over TIOLs in terms of CS, aberrations, and visual disturbance are not significant. Patients are satisfied with both types of IOLs.

## 1. Introduction

Lens extraction combined with implantation of intraocular lenses (IOLs), which is an established and effective procedure, improves visual quality after cataract surgery [1–3]. Monofocal IOLs can provide excellent distance visual acuity [4]. However, patients can only see objects clearly around the area of focus, and spectacles are required for near and intermediate vision. With the increasing demand for spectacle independence, different presbyopia-correcting IOLs, such as multifocal IOLs (MIOLs), are recommended for better intermediate and near visual acuity [5–7].

MIOLs incorporate two or three foci through a diffractive or refractive design [8]. Patients implanted with MIOLs have better intermediate and near visual acuity and higher spectacle independence than those implanted with monofocal IOLs [2]. However, although patients implanted with MIOLs can see objects at different distances, they still cannot have satisfying visual quality between the separate foci [9]. Besides, the distribution of light in multiple focal points reduces postoperative contrast sensitivity (CS) and increases the incidence rate of disturbing photic phenomena such as halos and glare [10, 11]. To combat this problem, extended depth of focus (EDOF) IOLs were introduced. They were designed to provide good vision across a continuous range of foci and decrease the rate of visual disturbances [12–14].

The design pattern of EDOF IOLs elongates the depth of focus from intermediate to far distances and forms a continuum of foci [15, 16]. There are several types of EDOF IOLs, including IOLs based on diffractive optics, nondiffractive optics, small aperture, and bioanalogic designs [16]. TECNIS Symfony, the most widely used type of EDOF IOL in clinical practice, is an example of a diffractive hydrophobic EDOF IOL that has an achromatic pattern, which can compensate for corneal chromatic aberration [12, 14–16]. Previous studies have demonstrated that the TECNIS Symfony IOL showed great safety with low rates of posterior capsule opacification (PCO) and adverse events [17, 18]. As this type of IOL has an aspheric anterior surface and a continuum of foci instead of separate foci, they are expected to decrease the incidence of photic phenomena such as halos [14]. Thus, EDOF IOLs are considered to bridge the gap between MIOLs and monofocal IOLs [16].

Although the original concept of the EDOF design shows great advantages, the specific clinical effect of this new type of IOL is still not very clear. Whether it can challenge the traditional MIOLs remains to be verified. Liu et al. conducted a meta-analysis to compare the implantation of TECNIS Symfony IOLs with the implantation of monofocal IOLs and trifocal IOLs (TIOLs); however, only six studies that compared Symfony IOLs and TIOLs were included, and monocular uncorrected visual acuity, monocular and binocular corrected visual acuity, refraction, and aberrations were not compared in their analysis [11]. After that meta-analysis was conducted, a lot of original studies that compared TECNIS Symfony IOLs and TIOLs were conducted. The purpose of the present study was to meta-analyze the results of these original studies and provide more evidence for comparing TECNIS Symfony IOLs and TIOLs from multiple perspectives.

## 2. Methods

### 2.1. Search Strategy

This systematic review and meta-analysis was conducted in accordance with the guidelines of the Preferred Reporting Items for Systematic Reviews and Meta-analyses Statement [19]. The protocol for this meta-analysis is registered with PROSPERO (CRD42020187618). PubMed, Cochrane Library, EMBASE, and ClinicalTrial.gov. were searched for relevant controlled studies published from January 2000 to March 2020. No language restrictions were applied. The following terms were used to search the databases: “extended depth of focus,” “extended range of vision,” “EDOF,” “Lenses, intraocular” [Mesh], “Intraocular lens,” “Intraocular lenses,” “Lens, Intraocular,” “Implantable Contact Lens,” “Contact Lens, Implantable,” “Lens, Implantable Contact,” “Lens implantation, intraocular” [Mesh], “Implantation, Intraocular Lens,” “Implantations, Intraocular Lens,” “Intraocular Lens Implantation,” “Intraocular Lens Implantations,” and “Lens Implantations, Intraocular.” The reference lists of key articles and relevant systematic reviews were searched manually to identify other potentially relevant studies. Two reviewers (Y. G. and Y. W.) conducted the search independently; a third reviewer (X. J.) was consulted in cases of disagreement.

### 2.2. Eligibility Criteria and Outcomes

All randomized controlled trials (RCTs) and nonrandomized controlled studies (NRCSs) that compared implantation of EDOF IOLs and TIOLs after cataract surgery were selected. Ongoing studies and studies on binocular blended implantation were excluded. Monocular and binocular uncorrected distance visual acuity (UDVA), uncorrected intermediate visual acuity (UIVA), uncorrected near visual acuity (UNVA), and defocus curves were defined as primary outcomes. Different defocus levels were introduced, from +1.00 to −4.00 diopters (D), in 0.50 D steps. Secondary outcomes included monocular and binocular corrected distance visual acuity (CDVA), distance corrected intermediate visual acuity (DCIVA), distance-corrected near visual acuity (DCNVA), refraction (including spherical equivalent refractive error, residual sphere, and residual astigmatism), spectacle independence, CS, aberrations, quality of vision and patient satisfaction, complications, and adverse events.

### 2.3. Risk of Bias Assessment and Data Extraction

The quality of RCTs was assessed using the Cochrane Collaboration Risk of Bias Tool [20]. Seven different aspects were evaluated according to the domains of the tool: random sequence generation, allocation concealment, blinding of participants and personnel, blinding of outcome assessment, incomplete outcome data, selective reporting, and other potential bias. Regarding the level of risk of bias, each item was rated as having a “low risk,” “high risk,” or “unclear risk.”

The Newcastle-Ottawa Scale (NOS) was used to assess the risk of bias of NRCSs [21]. Three aspects were assessed, namely: the selection of groups, the comparability between patients in different treatment arms, and outcome assessment. To analyze and quantify the risk of bias using the NOS, a star system is used. Each item can get at most one star in the “selection” and “outcome assessment” domains, and two stars at most in the “comparability” domain. The total number of stars ranges from 0 to 9; studies that score 0 to 3, 4 to 6, and 7 to 9 are considered low-, moderate-, and high-quality studies, respectively.

Two reviewers (Y. G. and Y. W.) independently extracted data from the included studies using a standard form. Discrepancies between the decisions of the two reviewers were resolved by consensus; a third reviewer (X. J.) was consulted when necessary. We extracted the characteristics of each study and details of the outcomes mentioned previously. For continuous variables, including visual acuity, defocus curves, refraction, and CS, we extracted the mean values and standard deviations (SD). For dichotomous variables, the number of events, such as spectacle independence, in each treatment group was extracted. For data that could not be merged, such as quality of vision and patient satisfaction, we only summarized and described the results. We contacted the authors of the studies for additional information by e-mail when necessary.

### 2.4. Data Synthesis and Statistical Analysis

The Review Manager 5.3 and Stata/SE 14.0 for Windows (StataCorp LP, USA) software programs were used to analyze the data. The *Q* test and *I*^2^ statistic were used to assess heterogeneity, and *I*^2^ > 50% was regarded as an indication of substantial heterogeneity [20]. If *I*^2^ was above 50%, the random-effects model was used; otherwise, we utilized the fixed-effects model. Mean deviation (MD, mean value of EDOF IOLs minus mean value of TIOLs) with 95% confidence interval (CI) was calculated for the continuous variables except CS, and risk ratio (RR) or risk deviation (RD) with 95% CI was calculated for categorical variables. RD was chosen when the number of events equaled zero. For CS, various test tools and different expressions were used in the studies; thus, we used a standard mean deviation (SMD). *P* < 0.05 was considered statistically significant. The publication bias of each study was checked using funnel plots and Egger's test. When a funnel plot asymmetry was noted, the trim-and-fill analysis was conducted to adjust the effect. Influence analysis was performed to verify the stability of the results.

For studies with two different TIOL implantation groups, the mean values and SDs for meta-analysis were combined according to formula (1) and formula (2), respectively. The total number of patients in the trifocal group is the sum of the two different trifocal groups. This data synthesis method was used by Cao et al. in a meta-analysis [2].

(1) $$\begin{array}{c} {\bar{x}}_{T}=\frac{n_{1}{\bar{x}}_{1}+n_{2}{\bar{x}}_{2}}{n_{1}+n_{2}}, \end{array}$$

(2) $$\begin{array}{c} {\text{SD}}_{T}=\sqrt{\frac{\left(n_{1}-1\right){\text{SD}}_{1}^{2}+\left(n_{2}-1\right){\text{SD}}_{2}^{2}+\left(n_{1}n_{2}/n_{1}+n_{2}\right){\left({\bar{x}}_{1}-{\bar{x}}_{2}\right)}^{2}}{n_{1}+n_{2}-1}}. \end{array}$$

## 3. Results

### 3.1. Search Results and Characteristics of the Included Studies

**Figure 1:**
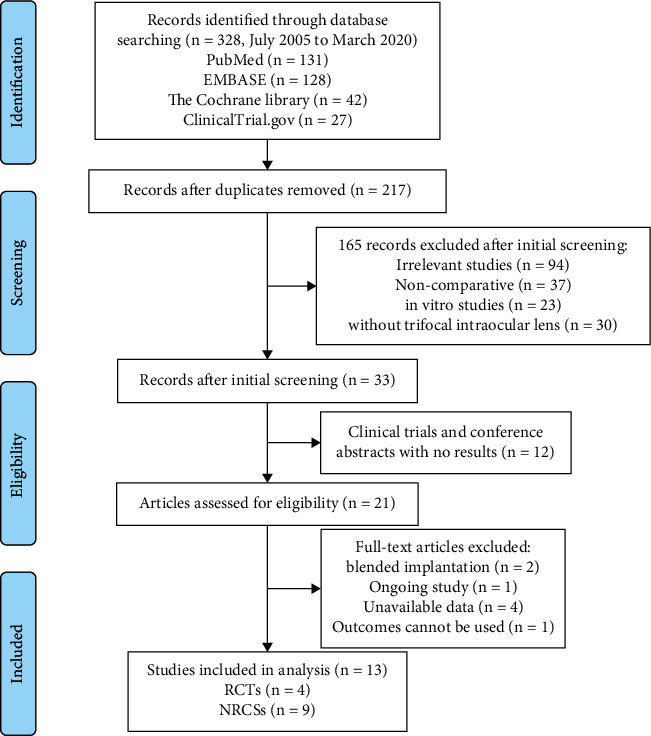
The flow diagram of identification and inclusion of the eligible studies based on the Preferred Reporting Items for Systematic Reviews and Meta-analyses flowchart.

As shown in Figure 1, a total of 328 records were identified after searching. Twenty-one records were considered eligible after the initial screening. After further consideration, two articles were excluded because they were studies on blended implantation [22, 23], and four studies were excluded because their specific data were not available [24–27], and one study was excluded because it was an ongoing clinical trial [28]. After reading the full text, one study was excluded because its outcome could not be included in the analysis [29]. Finally, 13 studies (four RCTs [30–33] and nine NRCSs [18, 34–41]) were included in the analysis.

The characteristics of the included studies are summarized in Table 1. These studies were conducted in different countries. All studies were published between 2017 and 2020. A total of 1,221 eyes were included in the analysis. The TECNIS Symfony ZXR00 IOL was implanted in the eyes included in the EDOF group, whereas AcrySof IQ PanOptix, FineVision Micro F, FineVision Pod F, and AT LISA tri 839MP IOLs were implanted in the eyes included in the trifocal group. The parameters of these included IOLs are listed in Table S1. The follow-up duration of the studies ranged from 1 to 29 months.

### 3.2. Assessment of Risk of Bias

**Figure 2:**
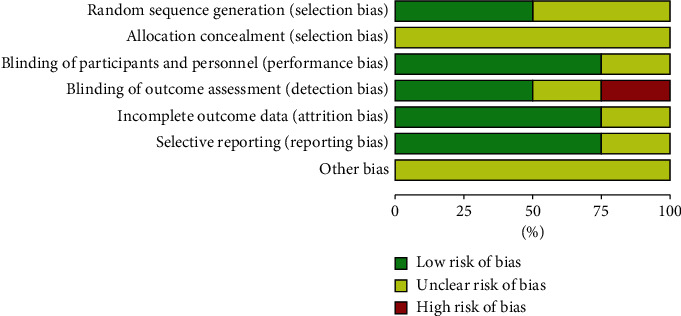
Risk of bias graph for the included randomized controlled trials.

**Figure 3:**
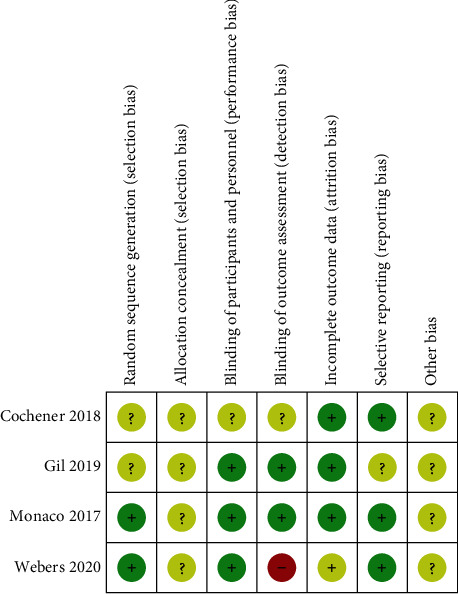
Summary of the risk of bias in the included randomized controlled trials.

The risks of bias in the RCTs are shown in Figures 2 and 3. In Weber's study, the randomization was disclosed at the three-month time point of the follow-up; this suggests the risk of detection bias. Details on allocation concealment were not clearly indicated in all studies, so the risk of bias is unclear in that regard. No other biases were found in all studies.

The qualities of the included NRCSs are shown in Table 2. Seven studies had 7–9 points, indicating that they are high-quality studies, whereas Rodov's and Lin's studies had 5 and 6 points, respectively, indicating that the studies are of moderate quality. Rodov's study is a retrospective study, and there were significant differences in parameters such as age and preoperative astigmatism in different groups. In Lin's study, no baseline information, such as patient age, was provided, and no long-term follow-up was conducted. Most studies had a sufficiently long follow-up period, and there was no significant difference in the baseline information of patients in different groups.

### 3.3. Primary Outcomes

#### 3.3.1. Monocular and Binocular Uncorrected Visual Acuity

**Figure 4:**
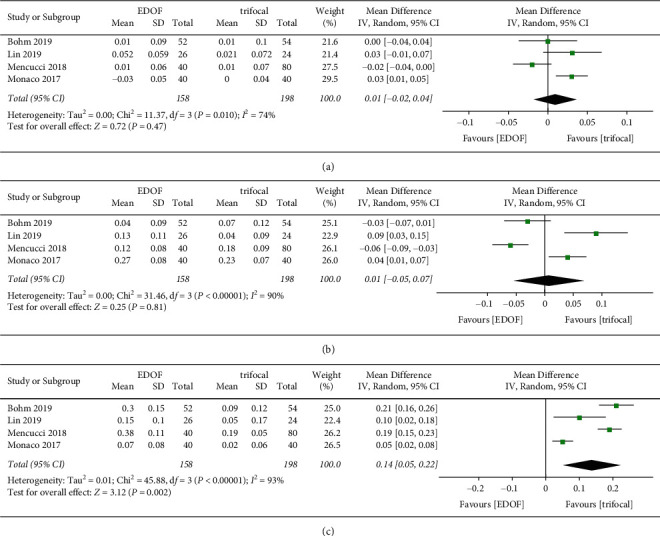
Forest plot of monocular uncorrected visual acuity. (a) Uncorrected distance visual acuity; (b) uncorrected intermediate visual acuity; and (c) uncorrected near visual acuity. EDOF, extended depth-of-focus; SD, standard deviation; CI, confidence interval; IV, inverse variance.

**Figure 5:**
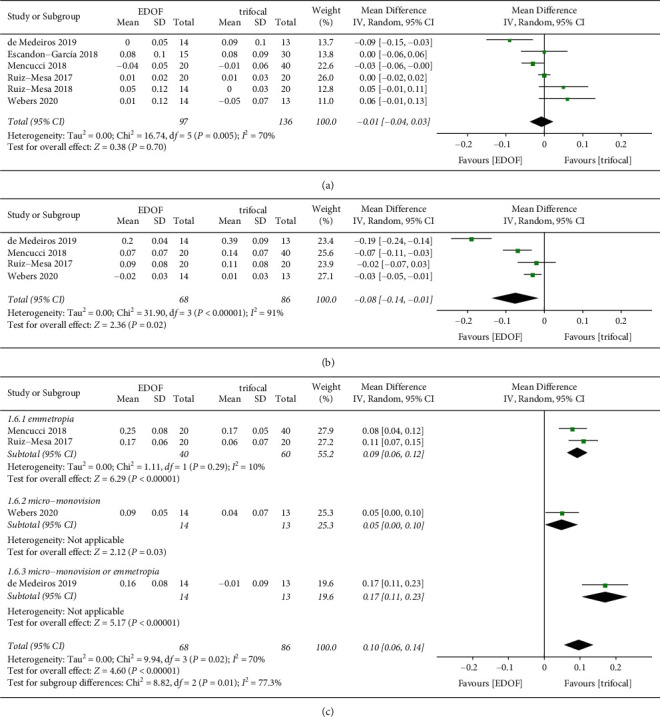
Forest plot of binocular uncorrected visual acuity. (a) Uncorrected distance visual acuity; (b) uncorrected intermediate visual acuity; and (c) uncorrected near visual acuity. EDOF, extended depth-of-focus; SD, standard deviation; CI, confidence interval; IV, inverse variance.

Visual acuity was reported in decimal format in two studies [30, 38]; we contacted the corresponding authors of the articles and received no responses. Therefore, data on uncorrected visual acuity were extracted from only 10 studies. Data on monocular uncorrected visual acuity were available in four studies, which included 158 eyes (Figure 4). There was no significant difference in monocular UDVA and UIVA between groups. Monocular UNVA was significantly worse in the EDOF group (MD: 0.14, 95% CI: 0.05 to 0.22, *P*=0.002, logMAR) than in the trifocal group. Seven, five, and five studies reported binocular UDVA, UIVA, and UNVA, respectively (Figure 5). There was no significant difference in binocular UDVA between groups. EDOF IOLs provided better binocular UIVA (MD: -0.08, 95% CI: −0.14 to −0.01, *P*=0.02, logMAR) but worse UNVA (MD: 0.10, 95% CI: 0.06 to 0.14, *P* < 0.0001, logMAR) than TIOLs. One study reported that binocular UNVA would be better if EDOF IOLs were targeted for micro-monovision (MD: 0.05, 95% CI: 0.00 to 0.10, *P*=0.03, logMAR) [32].

#### 3.3.2. Defocus Curves

**Figure 6:**
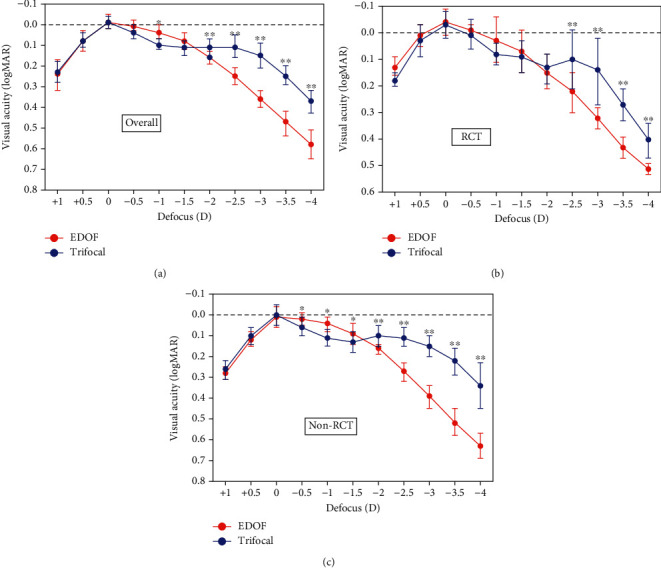
(a) The overall defocus curve; (b) the defocus curve synthesized from randomized controlled trials (RCTs); and (c) the defocus curve synthesized from prospective comparative studies. The bar represents the standard deviation. ^*∗*^*P* < 0.05, ^*∗∗*^*P* < 0.01. EDOF, extended depth-of-focus.

Figure 6 shows the defocus curves derived from all the included studies. The MDs of visual acuity (logMAR) at different defocus levels in EDOF IOLs and TIOLs are listed in Table S2. Overall, the EDOF group had statistically significantly better intermediate visual acuity at −1D (1 m) (MD: −0.06, 95% CI: −0.10 to −0.05, *P*=0.011) than the trifocal group, whereas the trifocal group performed significantly better at −2D to −4D (50 cm to 25 cm) than the EDOF group. A subgroup analysis was conducted based on study types (RCT or non-RCT). For non-RCTs, the EDOF group had statistically significantly better intermediate visual acuity from −0.5D to -1.5D (2 m to 67 cm) than the trifocal group; the MDs were −0.03, −0.06, and −0.04 (95% CI: −0.04, −0.01; −0.10, −0.01; −0.08, −0.00) for −0.5D, −1D, and −1.5D, respectively. For RCTs, the EDOF group also performed better at these defocus levels than the trifocal group, even without statistical differences. However, for near visual acuity, the trifocal group performed better than the EDOF group from −2.5D to −4D (40 cm to 25 cm).

### 3.4. Secondary Outcomes

#### 3.4.1. Monocular and Binocular Corrected Visual Acuity

**Figure 7:**
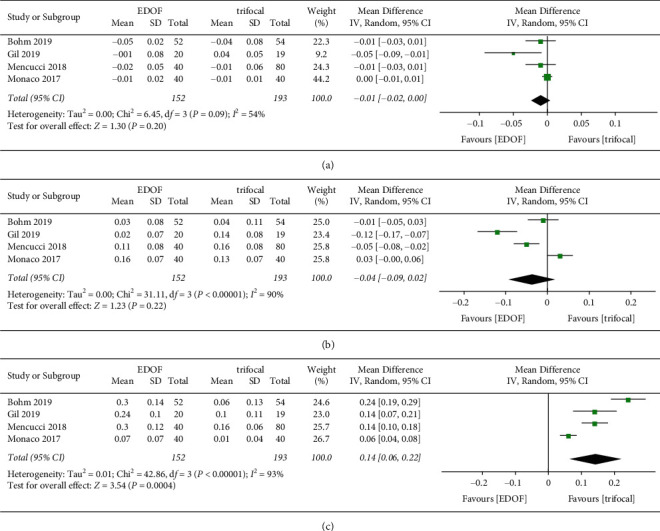
Forest plot of monocular corrected visual acuity. (a) Corrected distance visual acuity; (b) distance corrected intermediate visual acuity; and (c) distance corrected near visual acuity. EDOF, extended depth-of-focus; SD, standard deviation; CI, confidence interval; IV, inverse variance.

**Figure 8:**
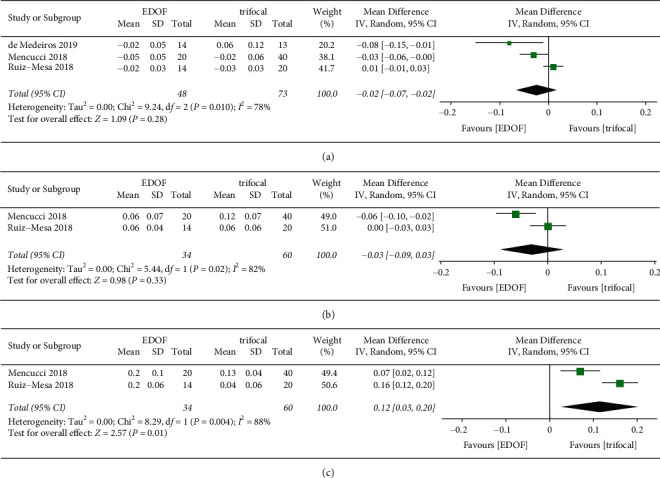
Forest plot of binocular corrected visual acuity. (a) Corrected distance visual acuity; (b) distance corrected intermediate visual acuity; and (c) distance corrected near visual acuity. EDOF, extended depth-of-focus; SD, standard deviation; CI, confidence interval; IV, inverse variance.

Monocular corrected visual acuity was reported in four studies (Figure 7). Three studies provided details on binocular CDVA, whereas two studies provided data on binocular DCIVA and DCNVA (Figure 8). There was no significant difference in monocular and binocular CDVA and DCIVA between groups. The EDOF group showed worse monocular (MD: 0.14, 95% CI: 0.06 to 0.22, *P*=0.0004, logMAR) and binocular (MD: 0.12, 95% CI: 0.03 to 0.20, *P*=0.01, logMAR) DCNVA than the trifocal group.

#### 3.4.2. Refraction

**Figure 9:**
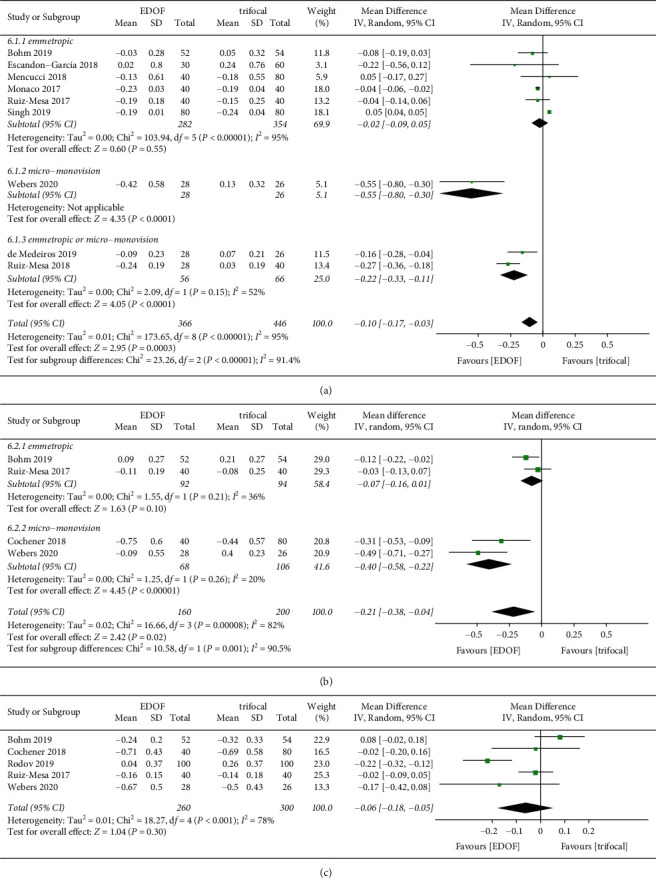
Forest plot of postoperative refraction. (a) Spherical equivalent; (b) residual sphere; and (c) residual astigmatism. EDOF, extended depth-of-focus; SD, standard deviation; CI, confidence interval; IV, inverse variance.

Spherical equivalent, residual sphere, and residual astigmatism were reported in nine, four, and five studies, respectively (Figure 9). There was no significant difference in spherical equivalent and residual sphere between groups in studies that targeted emmetropic eyes. There was also no significant difference in residual astigmatism between groups.

#### 3.4.3. Contrast Sensitivity

**Figure 10:**
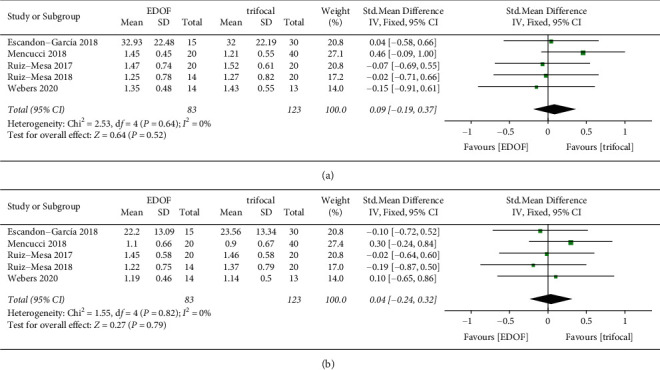
Forest plots of mean contrast sensitivity (CS). (a) The forest plot of CS under photopic conditions; and (b) the forest plot of CS under mesopic conditions. EDOF, extended depth-of-focus; SD, standard deviation; CI, confidence interval; IV, inverse variance.

The Functional Acuity Contrast Test was used to test CS in three studies [18, 35, 36], the Optec 6500 was used in one study [34], and the CSV-1000 system was used in one study [32]. There was no statistical difference in mean CS under photopic (SMD: 0.09, 95% CI: −0.19 to 0.37, *P*=0.52) and mesopic conditions (SMD: 0.04, 95% CI: −0.24 to 0.32, *P*=0.79) between the EDOF and trifocal groups (Figure 10); no heterogeneity was noted either. Overall, there was no statistical difference in CS from 1.5 cycles per degree (cpd) to 18 cpd between the two IOL groups, whether under photopic or mesopic conditions (Table S3). A subgroup analysis based on the testing methods for CS was conducted. The modulation transfer function (MTF) was used to reflect CS in three studies [30, 31, 38]. The MTF for EDOF IOLs and TIOLs was also reported to be comparable at different cpd and with 3 mm and 5 mm pupil diameters (PD).

#### 3.4.4. Aberrations

Aberrations were reported in five studies (Table 3). Quantitative analysis was not appropriate since different pupil sizes and different aberration types were measured in each study. Therefore, the results were summarized (Table 3). No significant difference was found in most studies, except in Monaco's study, in which patients in the EDOF group with a 5.0 mm PD had significantly higher values of intraocular aberrations and total aberrations than the patients in the trifocal group.

#### 3.4.5. Spectacle Independence

**Figure 11:**
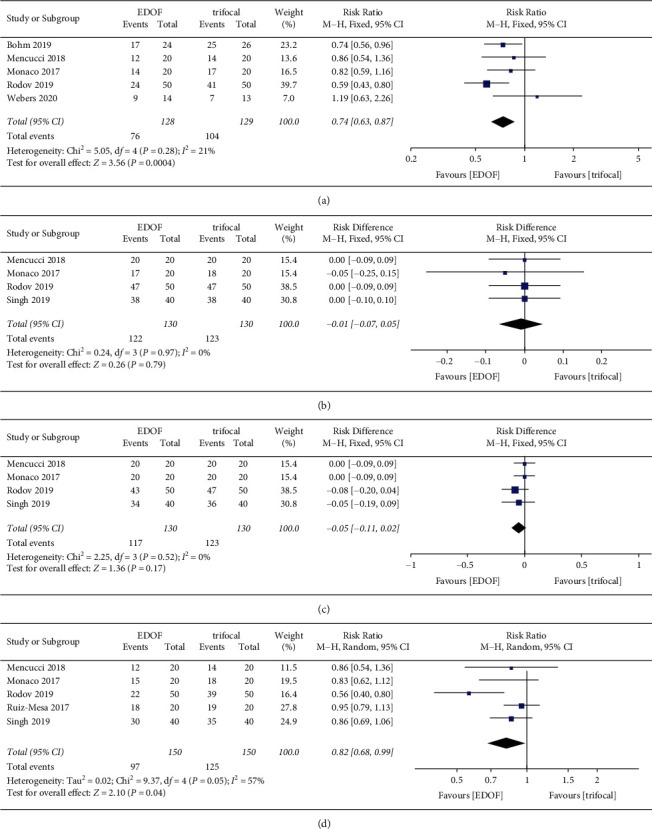
Forest plot of postoperative spectacle independence. (a) Total spectacle independence; (b) for distance vision; (c) for intermediate vision; and (d) for near vision. EDOF, extended depth-of-focus; CI, confidence interval.

Total spectacle independence was reported in five studies. Spectacle independence for distance and intermediate vision was reported in four studies, whereas spectacle independence for near vision was reported in five studies (Figure 11). The EDOF group had a significantly lower total spectacle independence (RR: 0.74, 95% CI: 0.63 to 0.87, *P*=0.0004) and a lower spectacle independence for near vision (RR: 0.82, 95% CI: 0.68 to 0.99, *P*=0.04) than the trifocal group. There was no significant difference in spectacle independence for distance and intermediate vision between the groups. The *I*^2^ was 0, indicating that there was no significant heterogeneity in different studies.

#### 3.4.6. Quality of Vision and Patient Satisfaction

Quality of vision and patient satisfaction were assessed in 11 studies through questionnaires. Patient satisfaction after implantation of both EDOF IOLs and TIOLs was high; 80–100% of patients were satisfied with TIOLs, whereas 90%–100% of patients were satisfied with EDOF IOLs [18, 34]. More than 75% of patients responded that they would choose the same lens again [18, 34, 39], whereas 92% with AT LISA tri 839MP IOL implants and 93% of patients with Symfony IOL implants would recommend the implanted IOL to family or friends [32].

Halos were reported as the most frequent, severe, and bothersome visual symptom in both groups [31]. Webers et al. also found that disabling glare (8% vs 7%; *P*=0.96) occurred less often than disabling halo (39% vs 21%, *P*=0.33) in the AT LISA tri 839MP and Symfony groups, respectively [32]. The frequency of the occurrence of halos and glare varied, ranging from less than 1% to 70% and 50%, respectively [30–32, 34]. Regarding halos and glare, there was no significant difference between the EDOF group and the trifocal group in most studies, except in Rodov's study, in which the proportion of patients who had postoperative halos or glare was significantly higher in the trifocal group than in the EDOF group (14% vs 38%, *p* < 0.001).

However, visual disturbance did not have a significant influence on patients [30, 34]. A well-validated questionnaire, the Quality of Vision (QoV) questionnaire [42], was used to assess visual disturbance in three studies [31, 36, 38]; a higher score in the QoV questionnaire means a worse quality of vision. In all studies, the mean QoV score was not statistically significant between groups [31, 38], except in the study by Escandón-García et al., in which patients implanted with Symfony IOLs showed higher values in all categories (frequency, severity, and bothersome) than patients implanted with TIOLs; the difference was statistically significant for the “bothersome” subscale. However, in the study by Gil et al., in which the Visual Function Index-14 questionnaire was used, the median score for life quality was significantly higher in the EDOF group than in the trifocal group (*P*=0.039), indicating that patients were more satisfied with the EDOF IOL.

#### 3.4.7. Complications and Adverse Events

Three studies reported postoperative complications and adverse events. In the study by Webers et al., one patient in both groups underwent neodymium:YAG laser capsulotomy treatment for PCO 3 months postoperatively [18]. In the study by Ruiz-Mesa et al. 5% of patients developed PCO in the Finevision group, while no patients had PCO in the EDOF group 12 months postoperatively [31]. Monaco et al. reported that there were no complications or adverse events in both groups of patients 4 months postoperatively [32].

### 3.5. Publication Bias

All indicators were tested by using Egger's test to assess publication bias (Table S4). Publication bias was found in spectacle independence for distance vision, visual acuity at a defocus level of +1D, and CS (1.5cpd, mesopic). We used the trim-and-fill method to adjust these indicators, after which the results remained unchanged (Table S5).

## 4. Discussion

In this study, we compared the outcomes of the implantation of TIOLs and TECNIS Symfony IOLs. The results indicated that the TECNIS Symfony IOLs provide significantly better intermediate vision, but worse near vision than TIOLs. Spectacle independence was found to be lower for near vision and for any purpose in the EDOF group than in the trifocal group. Distance visual acuity, CS, and aberrations were comparable between the two groups. Patient satisfaction did not differ between the two groups; most of the patients were satisfied with both IOLs.

A previous meta-analysis showed that there is no statistically significant difference between EDOF IOLs and TIOLs in terms of binocular UDVA [11], a finding which is consistent with our results. Furthermore, we found that distance visual acuity with the two types of IOL is comparable, whether monocular, binocular, uncorrected, or corrected. This result means that patients implanted with EDOF IOLs could have great distance visual acuity, even comparable with that of monofocal IOLs in previous studies [6, 7].

In *in vitro* experiments, EDOF technology provides clear and focused vision across near, intermediate, and distance ranges [43]. In population-based research, EDOF has been proven to perform better than monofocal IOLs at intermediate and near distances [6, 7]. Gatinel et al. measured and plotted through-focus MTF curves for 2, 3, and 3.75 mm pupil apertures; they discovered two MTF peaks for distance and intermediate vision with EDOF IOLs [44]. Correspondingly, the EDOF IOLs displayed a range of vision with sufficient resolution from 0 to +2D (from distance to 50 cm) and a sharp drop of contrast resolution at near distances [44]. The TIOLs displayed three MTF peaks for distance, intermediate, and near vision; however, the intermediate peak was relatively flat [44]. In the present study, patients implanted with EDOF IOLs had a significantly better binocular UIVA than those with TIOL implants; this is in contrast to the result of the previous meta-analysis, in which there was no difference in binocular UIVA between the two groups [11]. This difference may be due to the difference in the number of studies included in the meta-analyses.

EDOF IOLs provide patients with a better postoperative near visual acuity than monofocal IOLs [11]. However, the results of the present study indicate that they are still inferior to TIOLs. Spectacle independence for near vision was also significantly lower in the EDOF group than in the trifocal group in the present study. Recently, a new strategy called micro-monovision was proposed; it is reported to provide significantly better intermediate and near visual acuity than non-monovision [14]. In Weber's study, patients in the micro-monovision group underwent bilateral implantation of EDOF IOLs with the aim of making the dominant eye emmetropic and the nondominant eye -0.5D (mean refractive spherical equivalent). In the present study, the difference between binocular UNVA with EDOF IOLs targeted for micro-monovision and binocular UNVA with TIOLs was smaller than that between binocular UNVA with EDOF IOLs targeted for emmetropia and binocular UNVA with TIOLs. Moreover, under mesopic conditions, binocular UNVA with micro-monovision is better than that with TIOLs [32]. Tarib et al. applied a mix-and-match approach (the EDOF IOL in the dominant eye and the TIOL in the nondominant eye) to bilateral IOL implantation, and an improved near visual acuity, accompanied with a decreased intermediate visual acuity, was the outcome [22].

According to the defocus curves, the patients implanted with EDOF IOLs and TIOLs have comparable visual acuity from +1D to 0D, but EDOF IOLs provide better visual acuity from −0.5 D to −1.5D and worse visual acuity from −2.5D to −4D than TIOLs. Although not all differences were statistically significant, the results can still help surgeons select a suitable IOL to meet a patient's preferred visual acuity at specific distances. In the previous meta-analysis, no statistically significant difference was observed at a 1.0D defocus level, a finding which is in contrast to our results [11]. This may be due to the difference in the number of included articles. When patients demand a full range of vision from distance to near, or a specific visual quality for near tasks (e.g., reading), TIOLs are recommended. If a better intermediate visual quality (e.g., computer work) is required, the EDOF IOLs seem to be a satisfactory choice.

The results of the present study confirmed that near visual acuity with EDOF IOLs is inferior to that with TIOLs; however, this is not the case with reading performance. Most of the previous studies reported that patients implanted with EDOF IOLs and those implanted with MIOLs have a similar reading performance, including reading acuity, reading speed, and critical print size, under the photopic and mesopic conditions [13, 32, 34]. However, Ruiz-Mesa et al. reported a statistically significant worse visual acuity with EDOF IOLs than MIOLs at preferred reading distances, but the testing methods and luminance were not described in the study [35]. In *in vitro* experiments, the EDOF technique shows a satisfying tolerance to the presence of astigmatism and decentration [43, 45]. Overall, the worse near vision did not seem to affect patient satisfaction, which was high for both EDOF IOLs and TIOLs.

The EDOF IOL is designed with an aspheric anterior surface and a posterior achromatic diffractive surface to reduce the loss of CS, compensate for corneal chromatic aberration, and decrease visual disturbance [12, 14–16]. In the present study, there was no significant difference in CS between the EDOF and trifocal groups. Theoretically, multiple focal points will induce a lighter scatter than a single focal point, and forward scattering of light out of a glare source will generate a more veil of luminance on the retina. In addition, the out-of-focus image tends to have a larger diameter than the sharp image, which forms halos. Besides that, EDOF IOLs are also designed with a high negative spherical aberration, which is necessary to extend the depth of focus, but may also cause a perception of a larger halo. On the other hand, TIOLs are designed with a slightly negative spherical aberration, which can counterbalance corneal aberration and improve visual quality [36]. Therefore, for visual disturbance, the advantage of EDOF IOLs is not apparent. However, more studies are needed in the future to verify this conclusion.

In this meta-analysis, we systematically compared the visual performance of EDOF IOLs and TIOLs. To reduce the bias from the IOL type, we excluded bifocal IOLs from the analysis. However, as the review may be limited by the number of RCTs, NRCS were included as well. Further, we only included Symfony IOLs as a representative of EDOF IOLs because at the time of our database search, no RCTs or NRCSs that compared TIOLs and other types of EDOF IOLs were found. Thus, more clinical studies on the other EDOF IOLs are needed.

In conclusion, Symfony IOLs and TIOLs provide comparable distance vision. However, Symfony IOLs provide better intermediate vision and worse near vision than TIOLs. The advantages of Symfony IOLs over TIOLs in terms of CS, aberrations, and visual disturbance are not significant. Patients are satisfied with both types of IOLs. Our study provides more evidence for surgeons to select more suitable IOLs for cataract patients. In the future, designers can focus on the defects of these two types of IOLs more specifically to improve the design principles so that patients can obtain a higher visual quality.

## Figures and Tables

**Table 1:** Characteristics of the included studies (*n* = 13).

Study	Location	Design	EDOF			Trifocal			Follow-up duration (months)
			IOL types	*N* (patients/eyes)	Age (mean ± SD, years)	IOL types	*N* (patients/eyes)	Age (mean ± SD, years)	
Monaco et al. 2017 [31]	Italy	RCT	TECNIS Symfony	20/40	67.0 ± 8.5	AcrySof IQ PanOptix	20/40	66.0 ± 5.5	4
Ruiz-Mesa et al. 2017 [18]	Spain	NRCS	TECNIS Symfony	20/40	59.5 ± 8.9	FineVision Micro F	20/40	54.5 ± 7.2	12
Cochener et al. 2018 [30]	France	RCT	TECNIS Symfony	20/40	69.2 ± 8.4	AcrySof IQ PanOptix	20/40	62.5 ± 4.6	6
						FineVision Micro F	20/40	62.5 ± 4.6	
Mencucci et al. 2018 [34]	Italy	NRCS	TECNIS Symfony	20/40	68.9 ± 4.8	AcrySof IQ PanOptix	20/40	70.1 ± 4.8	3
						AT LISA tri 839MP	20/40	71.6 ± 4.4	
Ruiz-Mesa et al. 2018 [35]	Spain	NRCS	TECNIS Symfony	14/28	63.1 ± 10.0	AcrySof IQ PanOptix	20/40	63.8 ± 8.1	9∼29
Escandon-Garcia et al. 2018 [36]	Portugal	NRCS	TECNIS Symfony	15/30	63.5 ± 9.4	AcrySof IQ PanOptix	7/14	62.3 ± 9.0	1∼3
						FineVision Pod F	23/46	62.6 ± 8.0	
de Medeiros et al. 2019 [37]	Brazil	NRCS	TECNIS Symfony	14/28	NA	AcrySof IQ PanOptix	13/26	NA	6∼12
Singh et al. 2019 [38]	India	NRCS	TECNIS Symfony	40/80	69.1 ± 6.1	FineVision Micro F	40/80	66.1 ± 5.1	6
Rodov et al. 2019 [39]	Israel	NRCS	TECNIS Symfony	50/100	67.21 ± 9.83	FineVision Micro F	50/100	67.01 ± 6.73	1∼22
Böhm et al. [40]	Germany	NRCS	TECNIS Symfony	26/52	69.23 ± 8.17	AT LISA tri 839MP	27/54	63.51 ± 7.94	3
Gil et al. 2020 [33]	Spain	RCT	TECNIS Symfony	20/20	68.2 ± 6.2	AT LISA tri 839MP	19/19	68.7 ± 10.3	6
Lin et al. 2019 [41]	China	NRCS	TECNIS Symfony	26/26	NA	AT LISA tri 839MP	24/24	NA	1
Webers et al. 2020 [32]	Netherlands	RCT	TECNIS Symfony	14/28	67.57 ± 12.21	AT LISA tri 839MP	13/26	70.38 ± 6.08	3

IOL, intraocular lens; RCT, randomized controlled trial; NRCS, nonrandomized controlled study; EDOF, extended depth-of-focus

**Table 2:** The quality of the included nonrandomized controlled studies assessed with the Newcastle-Ottawa Scale.

Study	Selection				Comparability	Outcome assessment			Total quality score
	Representativeness of the treatment arm	Selection of the comparative treatment arm(s)	Ascertainment of treatment regimen	Outcome was not present at the start of the study	Comparability between patients in different treatment arms: age, preoperative conditions	Assessment of outcome with independency or with records	Adequacy of follow-up duration (more than three months)	Lost to follow-up (acceptable - less than 10%)	
Ruiz-Mesa et al. 2017 [18]	☆	☆	☆	☆	☆	☆	☆	☆	8
Mencucci et al. 2018 [34]	☆	☆	☆	☆	☆☆	☆	☆	☆	9
Ruiz-Mesa et al. 2018 [35]	☆	☆	☆	☆	☆	☆	☆	☆	8
Escandon-Garcia et al. 2018 [36]	☆	☆	☆	☆	☆☆	☆		☆	8
de Medeiros et al. 2019 [37]	☆	☆	☆	☆	☆☆	☆	☆	☆	9
Singh et al. 2019 [38]	☆	☆	☆	☆	☆☆	☆	☆	☆	9
Rodov et al. 2019 [39]	☆	☆	☆			☆		☆	5
Bohm 2019	☆	☆	☆	☆	☆	☆	☆	☆	8
Lin et al. 2019 [41]	☆	☆	☆	☆		☆		☆	6

Each item can get at most one star (☆) in “selection” and “outcome assessment,” and two stars (☆☆) at most in “comparability.” The total number of stars ranges from 0 to 9; studies that score 0 to 3, 4 to 6, and 7 to 9 are considered have a low, moderate, and high quality, respectively.

**Table 3:** Summary of aberrations.

Study	EDOF	Trifocal	Device	Results
Cochener et al. 2018 [30]	TECNIS Symfony	AcrySof IQ PanOptix FineVision Micro F	iTrace	With a 4.0 mm PD, there was no significant difference in HOAs, coma, tilt, and spherical aberrations between groups.
Monaco et al. 2017 [31]	TECNIS Symfony	AcrySof IQ PanOptix	OPD-Scan II	Intraocular aberrations: (i) With a 3.0 mm PD, primary spherical aberration was significantly higher with the EDOF IOL than with the trifocal IOL. (ii) With a 5.0 mm PD, the RMS of HOAs was significantly higher with the EDOF IOL than with the trifocal IOL. Primary spherical aberration was also significantly higher with the EDOF IOL than with the trifocal IOL Total aberrations: (i) With a 3.0 mm PD, total aberrations with the EDOF IOL and the trifocal did not differ statistically. (ii) With a 5.0 mm PD, the RMS of LOAs, HOAs, and coma was higher in the EDOF group than in the trifocal group. Primary spherical aberration was higher in the EDOF group than in the trifocal group. There were no statistically significant differences in Strehl ratios between groups.
Ruiz-Mesa et al. 2018 [35]	TECNIS Symfony	AcrySof IQ PanOptix	iTrace	With a 3.0 mm PD, there was no significant difference in RMS of HOA between groups.
Singh et al. 2019 [38]	TECNIS Symfony	FineVision Micro F	OPD-Scan II	There was no significant difference in Strehl ratios of PSF between groups with both 3.0 mm and 5.0 mm PD.
Lin et al. 2019 [41]	TECNIS Symfony	AT LISA tri 839MP	OPD-scan III	There was no significant difference in total, tilt, high, coma, trefoil, and spherical aberrations between groups with 3.0 mm, 4.0 mm, and 5.0 mm PDs.

PD, pupil diameter; RMS, root mean square; HOAs, higher order aberrations; LOAs, lower order aberrations; PSF, point-spread function; IOL, intraocular lens; EDOF, extended depth of focus

## Data Availability

The datasets supporting the conclusions of this article are all included within the article and its additional file.

## Supplementary Materials

Supplementary Materials Table 1S: parameters of the included intraocular lenses. Table 2Sa: overall mean values and 95% CI of visual acuity (logMAR) at different defocus. Table 2Sb: mean values and 95% CI of visual acuity (logMAR) at different defocus from RCTs. Table 2Sc: mean values and 95% CI of visual acuity (logMAR) at different defocus from non-RCTs. Table 2Sd: MD and 95% CI of visual acuity (logMAR) at different defocus across EDOF IOLs and TIOLs. Table 3S: contrast sensitivity difference between EDOF IOLs and TIOLs. Table 4S. Egger's test for each outcome. Table 5S: trim-and-fill method for the adjustment of publication bias.
