# Supplementary material for: Comparison of Patient Outcomes following Implantation of Trifocal and Extended Depth of Focus Intraocular Lenses: A Systematic Review and Meta-Analysis
Source: J Ophthalmol. 2021 Dec 29;2021:1115076. doi: 10.1155/2021/1115076 (PMC8731298; doi:10.1155/2021/1115076)
Supplement: Supplementary Materials — Table 1S: parameters of the included intraocular lenses. Table 2Sa: overall mean values and 95% CI of visual acuity (logMAR) at different defocus. Table 2Sb: mean values and 95% CI of visual acuity (logMAR) at different defocus from RCTs. Table 2Sc: mean values and 95% CI of visual acuity (logMAR) at different defocus from non-RCTs. Table 2Sd: MD and 95% CI of visual acuity (logMAR) at different defocus across EDOF IOLs and TIOLs. Table 3S: contrast sensitivity difference between EDOF IOLs and TIOLs. Table 4S. Egger's test for each outcome. Table 5S: trim-and-fill method for the adjustment of publication bias. [file 1115076.f1.docx]

**Supplementary Materials**

**Supplementary 1**

**Table 1S. Parameters of the included intraocular lenses**

| Intraocular lenses | TECNIS Symfony ZXR00 | AcrySof IQ PanOptix | FineVision Micro F | FineVision Pod F | AT LISA tri 839 MP |
| --- | --- | --- | --- | --- | --- |
| Material | UV-filtering hydrophobic acrylic | methacrylate copolymer | 26% hydrophilic acrylic | 26% hydrophilic acrylic | hydrophilic copolymer with hydrophobized surface |
| Technology | Extended depth-of-focus | Trifocal | Trifocal | Trifocal | Trifocal |
| Diffractive area | 5.50 mm | 4.50 mm | 6.15 mm | 6.00 mm | 6.00 mm |
| Optic diameter | 6.00 mm | 6.00 mm | 6.15mm | 6.00mm | 6.00 mm |
| Overall diameters | 13 mm | 13 mm | 10.75mm | 11.4mm | 11mm |
| Geometry of central zone | Aspheric anterior surface & posterior achromatic diffractive surface | Diffractive | Diffractive | Diffractive aspheric front surface & posterior aspheric surface | Diffractive |
| Intermediate addition powers | - | +2.17D | +1.75D | +1.75D | +1.66D |
| Near addition powers | - | +3.25D | +3.50D | +3.50D | +3.33D |
| Spherical aberration | -0.27µm | -0.10µm | -0.11µm | -0.11µm | -0.18µm |
| Diopter range (increments) | +5.0-+34.0D (0.5D) | +13.0-+30.0D (0.5D)  +31.0-+34.0D (1.0D) | +10.0-+35.0D (0.5D) | +6.0-+35.0D (0.5D) | 0.0-+32.0D (0.5D) |

**Supplementary 2**

**Table 2Sa. Overall mean values and 95% CI of visual acuity (logMAR) at different defocus**

| Defocus | EDOF | | | | | Trifocal | | | | |
| --- | --- | --- | --- | --- | --- | --- | --- | --- | --- | --- |
|  | mean | 95%CI | | Model | I^2^ (%) | mean | 95%CI | | Model | I^2^ (%) |
| +1D | 0.24 | 0.17 | 0.32 | Random | 91.4 | 0.23 | 0.18 | 0.28 | Random | 90.1 |
| +0.5D | 0.08 | 0.03 | 0.13 | Random | 90.4 | 0.08 | 0.04 | 0.11 | Random | 89.1 |
| 0D | -0.01 | -0.05 | 0.02 | Random | 87.0 | -0.01 | -0.04 | 0.02 | Random | 94.3 |
| -0.5D | 0.01 | -0.02 | 0.03 | Random | 74.7 | 0.04 | 0.00 | 0.07 | Random | 92.0 |
| -1D | 0.04 | 0.00 | 0.07 | Random | 90.6 | 0.10 | 0.07 | 0.12 | Random | 83.5 |
| -1.5D | 0.08 | 0.04 | 0.12 | Random | 92.3 | 0.11 | 0.08 | 0.15 | Random | 89.8 |
| -2D | 0.16 | 0.13 | 0.19 | Random | 77.6 | 0.11 | 0.07 | 0.16 | Random | 95.3 |
| -2.5D | 0.25 | 0.21 | 0.29 | Random | 82.4 | 0.11 | 0.05 | 0.16 | Random | 95.3 |
| -3D | 0.36 | 0.32 | 0.40 | Random | 82.2 | 0.15 | 0.09 | 0.21 | Random | 95.3 |
| -3.5D | 0.47 | 0.42 | 0.54 | Random | 91.5 | 0.25 | 0.20 | 0.29 | Random | 90.0 |
| -4D | 0.58 | 0.51 | 0.65 | Random | 93 | 0.37 | 0.32 | 0.43 | Random | 94.5 |

CI, confidence interval; EDOF, extended depth of focus.

**Table 2Sb. Mean values and 95% CI of visual acuity (logMAR) at different defocus from RCTs**

| Defocus | EDOF | | | | | Trifocal | | | | |
| --- | --- | --- | --- | --- | --- | --- | --- | --- | --- | --- |
|  | mean | 95%CI | | Model | I^2^ (%) | mean | 95%CI | | Model | I^2^ (%) |
| +1D | 0.13 | 0.09 | 0.16 | Fixed | 0 | 0.18 | 0.15 | 0.20 | Fixed | 0 |
| +0.5D | 0.01 | -0.03 | 0.05 | Random | 58.9 | 0.03 | -0.03 | 0.09 | Random | 83.0 |
| 0D | -0.04 | -0.09 | 0.01 | Random | 86.9 | -0.03 | -0.08 | 0.02 | Random | 91.5 |
| -0.5D | -0.01 | -0.03 | 0.01 | Fixed | 32.6 | 0.01 | -0.05 | 0.06 | Random | 91.3 |
| -1D | 0.03 | -0.06 | 0.11 | Random | 95.6 | 0.08 | 0.04 | 0.12 | Random | 79.4 |
| -1.5D | 0.07 | -0.01 | 0.15 | Random | 95.9 | 0.09 | 0.03 | 0.15 | Random | 91.8 |
| -2D | 0.15 | 0.08 | 0.21 | Random | 90.7 | 0.13 | 0.08 | 0.19 | Random | 90.6 |
| -2.5D | 0.22 | 0.15 | 0.30 | Random | 91.2 | 0.10 | -0.01 | 0.21 | Random | 96.6 |
| -3D | 0.32 | 0.28 | 0.36 | Random | 69.2 | 0.14 | 0.02 | 0.27 | Random | 97.7 |
| -3.5D | 0.43 | 0.39 | 0.47 | Random | 60.5 | 0.27 | 0.21 | 0.33 | Random | 88.9 |
| -4D | 0.51 | 0.49 | 0.53 | Fixed | 45.5 | 0.40 | 0.34 | 0.47 | Random | 92.4 |

CI, confidence interval; RCT, randomized clinical trial; EDOF, extended depth of focus.

**Table 2Sc. Mean values and 95% CI of visual acuity (logMAR) at different defocus from non-RCTs**

| Defocus | EDOF | | | | | Trifocal | | | | |
| --- | --- | --- | --- | --- | --- | --- | --- | --- | --- | --- |
|  | mean | 95%CI | | Model | I^2^ (%) | mean | 95%CI | | Model | I^2^ (%) |
| +1D | 0.28 | 0.25 | 0.31 | Fixed | 47.3 | 0.26 | 0.22 | 0.31 | Random | 81.4 |
| +0.5D | 0.12 | 0.08 | 0.15 | Random | 67.1 | 0.10 | 0.06 | 0.14 | Random | 87.8 |
| 0D | 0.01 | -0.04 | 0.06 | Random | 89.3 | 0.00 | -0.05 | 0.05 | Random | 96.3 |
| -0.5D | 0.02 | -0.01 | 0.05 | Random | 82.3 | 0.06 | 0.01 | 0.10 | Random | 91.2 |
| -1D | 0.04 | 0.01 | 0.08 | Random | 82.9 | 0.11 | 0.07 | 0.15 | Random | 87.1 |
| -1.5D | 0.09 | 0.04 | 0.14 | Random | 89.5 | 0.13 | 0.08 | 0.18 | Random | 88.8 |
| -2D | 0.16 | 0.14 | 0.19 | Fixed | 40.5 | 0.10 | 0.05 | 0.15 | Random | 92.3 |
| -2.5D | 0.27 | 0.23 | 0.32 | Random | 67.1 | 0.11 | 0.06 | 0.15 | Random | 91.0 |
| -3D | 0.39 | 0.34 | 0.45 | Random | 75.3 | 0.15 | 0.10 | 0.20 | Random | 85.5 |
| -3.5D | 0.52 | 0.45 | 0.58 | Random | 77.9 | 0.22 | 0.16 | 0.29 | Random | 90.2 |
| -4D | 0.63 | 0.57 | 0.69 | Random | 79.0 | 0.34 | 0.23 | 0.45 | Random | 96.2 |

CI, confidence interval; RCT, randomized clinical trial; EDOF, extended depth of focus.

**Table 2Sd. MD and 95% CI of visual acuity (logMAR) at different defocus across EDOF IOLs and TIOLs**

| Defocus | Overall | | | RCT | | | Non-RCT | | |
| --- | --- | --- | --- | --- | --- | --- | --- | --- | --- |
|  | MD (95% CI) | *P* | I_2_ (%) | MD (95% CI) | *P* | I_2_ (%) | MD (95% CI) | *P* | I_2_ (%) |
| +1D | -0.00  (-0.05 ,0.05) | 0.971 | 70.5 | **-0.05***  **(-0.09, -0.01)** | 0.013 | 0 | 0.03  (-0.05, 0.11) | 0.403 | 75.8 |
| +0.5D | 0.01  (-0.02, 0.04) | 0.661 | 60.0 | -0.02  (-0.12, 0.08) | 0.692 | 87.4 | 0.01  (-0.02, 0.04) | 0.063 | 0 |
| 0D | -0.00  (-0.03, 0.02) | 0.871 | 70.5 | -0.02  (-0.07, 0.04) | 0.502 | 82.4 | 0.01  (-0.00, 0.03) | 0.107 | 0 |
| -0.5D | -0.02  (-0.05, 0.00) | 0.067 | 65.9 | -0.03  (-0.09, 0.04) | 0.430 | 84.4 | **-0.03***  **(-0.04, -0.01)** | 0.014 | 26.3 |
| -1D | **-0.06***  **(-0.10, -0.05)** | 0.011 | 86.4 | -0.06  (-0.15, 0.04) | 0.236 | 92.8 | **-0.06***  **(-0.10, -0.01)** | 0.014 | 77.5 |
| -1.5D | -0.03  (-0.09, 0.03) | 0.327 | 92.2 | -0.02  (-0.16, 0.11) | 0.760 | 96.8 | **-0.04***  **(-0.08, -0.00)** | 0.035 | 56.8 |
| -2D | **0.05****  **(0.02, 0.08)** | 0.002 | 58.3 | 0.02  (-0.01, 0.04) | 0.135 | 0 | **0.08****  **(0.05, 0.11)** | <0.001 | 0 |
| -2.5D | **0.15****  **(0.10, 0.20)** | <0.001 | 84.9 | **0.12****  **(0.06, 0.17)** | <0.001 | 67.2 | **0.17****  **(0.10, 0.24)** | <0.001 | 81.0 |
| -3D | **0.21****  **(0.15, 0.28)** | <0.001 | 89.7 | **0.17****  **(0.08, 0.27)** | 0.001 | 90.1 | **0.26****  **(0.23, 0.29)** | <0.001 | 32.5 |
| -3.5D | **0.23****  **(0.15, 0.31)** | <0.001 | 91.9 | **0.17****  **(0.08, 0.25)** | <0.001 | 84.1 | **0.29****  **(0.25, 0.32)** | <0.001 | 0 |
| -4D | **0.21****  **(0.12, 0.29)** | <0.001 | 92.7 | **0.12****  **(0.04, 0.20)** | 0.003 | 80.9 | **0.28****  **(0.22, 0.33)** | <0.001 | 56.8 |

MD, mean difference; CI, confidence interval; I2 = extent of inconsistency; EDOF, extended depth of focus; TIOLs, trifocal intraocular lenses; RCT, randomized controlled trial. * P<0.05, ** P<0.01.

**Supplementary 3**

**Table 3S. Contrast sensitivity difference between EDOF IOLs and TIOLs**

| Indicators | SMD | 95% CI | | I^2^ (%) | Model |
| --- | --- | --- | --- | --- | --- |
|  |  | Lower limit | Upper limit |  |  |
| Photopic condition | | | | | |
| 1.5cpd  FACT (n=3)  OPD (n=1) | 0.56  0.13  **1.88** | -0.33  -0.24  **1.24** | 1.45  0.50  **2.51** | 87  0  - | Random  Fixed  - |
| 3cpd  FACT (n=3)  OPD (n=1)  CSV-1000 (n=1) | 0.11  -0.05  1.10  -0.47 | -0.46  -0.42  0.53  -1.24 | 0.68  0.32  1.67  0.30 | 74.4  0  -  - | Random  Fixed  -  - |
| 6cpd  FACT (n=3)  OPD (n=1)  CSV-1000 (n=1) | -0.06  -0.21  **1.08**  -0.81 | -0.70  -0.58  **0.51**  -1.60 | 0.59  0.16  **1.65**  -0.03 | 79.6  0  -  - | Random  Fixed  -  - |
| 12cpd  FACT (n=3)  OPD (n=1)  CSV-1000 (n=1) | 0.08  -0.11  0.53  0.03 | -0.20  -0.48  -0.01  -0.72 | 0.36  0.26  1.08  0.79 | 8.1  0  -  - | Fixed  Fixed  -  - |
| 18cpd  FACT (n=2)  OPD (n=1)  CSV-1000 (n=1) | 0.33  -0.04  **1.21**  0.11 | -0.33  -0.50  **0.63**  -0.64 | 0.99  0.42  **1.79**  0.87 | 75.0  0  -  - | Random  Fixed  -  - |
| Mesopic condition | | | | | |
| 1.5cpd  FACT (n=3)  OPD (n=1) | 0.02  -0.23  **0.75** | -0.55  -0.61  **0.20** | 0.58  0.14  **1.31** | 69.8  0  - | Random  Fixed  - |
| 3cpd  FACT (n=3)  OPD (n=1)  CSV-1000 (n=1) | 0.06  -0.36  **1.70**  -0.31 | -0.84  -0.91  **1.08**  -1.07 | 0.95  0.19  **2.32**  0.45 | 88.9  53.0  -  - | Random  Random  -  - |
| 6cpd  FACT (n=3)  OPD (n=1)  CSV-1000 (n=1) | -0.17  **-0.43**  0.43  0.04 | -0.62  **-0.80**  -0.12  -0.71 | 0.29  **-0.05**  0.97  0.80 | 59.5  38.0  -  - | Random  Fixed  -  - |
| 12cpd  FACT (n=3)  OPD (n=1)  CSV-1000 (n=1) | 0.24  -0.03  **0.69**  0.54 | -0.04  -0.40  **0.14**  -0.23 | 0.53  0.34  **1.24**  1.31 | 49.5  26.6  -  - | Fixed  Fixed  -  - |
| 18cpd  FACT (n=1)  OPD (n=1)  CSV-1000 (n=1) | 0.20  -0.42  **0.59**  0.39 | -0.42  -1.11  **0.04**  -0.38 | 0.82  0.27  **1.14**  1.15 | 61.7  -  -  - | Random  -  -  - |

EDOF, extended depth-of-focus; IOL, intraocular lenses; TIOL, trifocal intraocular lenses; SMD, standard mean difference; CI, confidence interval; cpd, cycles per degree

**Supplementary 4**

**Table 4S. Egger’s test for each outcome**

| Indicators | t | df | P |
| --- | --- | --- | --- |
| Monocular UDVA-MD | -0.22 | 3 | 0.848 |
| Monocular UIVA-MD | 0.96 | 3 | 0.439 |
| Monocular UNVA-MD | 0.49 | 3 | 0.675 |
| Binocular UDVA-MD | 0.03 | 5 | 0.979 |
| Binocular UIVA-MD | -1.18 | 3 | 0.359 |
| Binocular UNVA-MD | 1.04 | 3 | 0.407 |
| Monocular CDVA-MD | -3.76 | 3 | 0.064 |
| Monocular DCIVA-MD | -1.35 | 3 | 0.310 |
| Monocular DCNVA-MD | 2.08 | 3 | 0.174 |
| Binocular CDVA-MD | -1.88 | 2 | 0.312 |
| Binocular DCIVA-MD | - | 1 | - |
| Binocular DCNVA-MD | - | 1 | - |
| Spherical equivalent-MD | -2.09 | 8 | 0.075 |
| Residual sphere-MD | -3.44 | 3 | 0.075 |
| Residual astigmatism-MD | -0.36 | 4 | 0.745 |
| Spectacle independence (any purpose)-RR | 1.78 | 4 | 0.174 |
| Spectacle independence for distance vision-RD | -28.90 | 3 | **0.001*** |
| Spectacle independence for intermediate vision-RD | -1.67 | 3 | 0.237 |
| Spectacle independence for near vision-RR | -1.39 | 4 | 0.259 |
| Defocus (+1D)-MD | 2.98 | 5 | **0.041*** |
| Defocus (+0.5D)-MD | -0.12 | 5 | 0.911 |
| Defocus (0D)-MD | -0.47 | 6 | 0.658 |
| Defocus (-0.5D)-MD | -0.35 | 6 | 0.738 |
| Defocus (-1D)-MD | 0.04 | 6 | 0.967 |
| Defocus (-1.5D)-MD | -0.38 | 6 | 0.723 |
| Defocus (-2D)-MD | 0.13 | 6 | 0.902 |
| Defocus (-2.5D)-MD | 0.32 | 6 | 0.758 |
| Defocus (-3D)-MD | 1.48 | 6 | 0.199 |
| Defocus (-3.5D)-MD | 1.47 | 5 | 0.215 |
| Defocus (-4D)-MD | 1.30 | 5 | 0.262 |
| Mean CS (Photopic, luminance≥80cpd)-SMD | -2.80 | 4 | 0.068 |
| CS (1.5cpd, Photopic, luminance≥80cpd)-SMD | -0.21 | 3 | 0.855 |
| CS (3cpd, Photopic, luminance≥80cpd) -SMD | -2.38 | 4 | 0.098 |
| CS (6cpd, Photopic, luminance≥80cpd) -SMD | -2.69 | 4 | 0.075 |
| CS (12cpd, Photopic, luminance≥80cpd) -SMD | -1.29 | 4 | 0.286 |
| CS (18cpd, Photopic, luminance≥80cpd) -SMD | -1.00 | 3 | 0.423 |
| Mean CS (Mesopic, luminance<10cpd) -SMD | -1.19 | 4 | 0.318 |
| CS (1.5cpd, Mesopic, luminance<10cpd) -SMD | -4.77 | 3 | **0.041*** |
| CS (3cpd, Mesopic, luminance<10cpd) -SMD | -1.16 | 4 | 0.331 |
| CS (6cpd, Mesopic, luminance<10cpd) -SMD | -1.46 | 4 | 0.240 |
| CS (12cpd, Mesopic, luminance<10cpd) -SMD | -0.48 | 4 | 0.663 |
| CS (18cpd, Mesopic, luminance<10cpd) -SMD | -0.60 | 2 | 0.655 |

UDVA, uncorrected distance visual acuity; UIVA, uncorrected intermediate visual acuity; UNVA, uncorrected near visual acuity; CDVA, corrected distance visual acuity; DCIVA, distance-corrected intermediate visual acuity; DCNVA, distance-corrected near visual acuity; CS, contrast sensitivity; MD, mean difference; RR, risk ratio; RD, risk deviation; SMD, standard mean difference; cpd, cycles per degree. * *P*<0.05.

**Supplementary 5**

**Table 5S. Trim-and-fill method for the adjustment of publication bias**

| Indicators | Model | Before adjusting | | After adjusting | |
| --- | --- | --- | --- | --- | --- |
|  |  | PE | 95% CI | PE | 95% CI |
| Spectacle independence for distance vision | Fixed  **random** | -0.003  -0.003 | -0.055, 0.049  -0.055, 0.049 | -0.005  -0.005 | -0.050, 0.041  -0.050, 0.041 |
| Defocus (+1D) | Fixed  **random** | 0.918  0.968 | 0.735, 1.147  0.621, 1.510 | 0.918  0.968 | 0.735, 1.147  0.621, 1.510 |
| CS (1.5cpd, Mesopic) | Fixed  **random** | 1.074  1.017 | 0.789, 1.462  0.578, 1.790 | 1.074  1.017 | 0.789, 1.462  0.578, 1.790 |

PE, pooled estimate; CI, confidence interval; CS, contrast sensitivity.
